# The Use of Biologically Related Model (Eclipse) for the Intensity-Modulated Radiation Therapy Planning of Nasopharyngeal Carcinomas

**DOI:** 10.1371/journal.pone.0112229

**Published:** 2014-11-05

**Authors:** Monica W. K. Kan, Lucullus H. T. Leung, Peter K. N. Yu

**Affiliations:** 1 Department of Oncology, Princess Margaret Hospital, Hong Kong SAR, China; 2 Department of Physics and Materials Science, City University of Hong Kong, Tat Chee Avenue, Kowloon Tong, Hong Kong SAR, China; West Virginia University School of Medicine, United States of America

## Abstract

**Purpose:**

Intensity-modulated radiation therapy (IMRT) is the most common treatment technique for nasopharyngeal carcinoma (NPC). Physical quantities such as dose/dose-volume parameters are used conventionally for IMRT optimization. The use of biological related models has been proposed and can be a new trend. This work was to assess the performance of the biologically based IMRT optimization model installed in a popular commercial treatment planning system (Eclipse) as compared to its dose/dose volume optimization model when employed in the clinical environment for NPC cases.

**Methods:**

Ten patients of early stage NPC and ten of advanced stage NPC were selected for this study. IMRT plans optimized using biological related approach (BBTP) were compared to their corresponding plans optimized using the dose/dose volume based approach (DVTP). Plan evaluation was performed using both biological indices and physical dose indices such as tumor control probability (TCP), normal tissue complication probability (NTCP), target coverage, conformity, dose homogeneity and doses to organs at risk. The comparison results of the more complex advanced stage cases were reported separately from those of the simpler early stage cases.

**Results:**

The target coverage and conformity were comparable between the two approaches, with BBTP plans producing more hot spots. For the primary targets, BBTP plans produced comparable TCP for the early stage cases and higher TCP for the advanced stage cases. BBTP plans reduced the volume of parotid glands receiving doses of above 40 Gy compared to DVTP plans. The NTCP of parotid glands produced by BBTP were 8.0±5.8 and 7.9±8.7 for early and advanced stage cases, respectively, while those of DVTP were 21.3±8.3 and 24.4±12.8, respectively. There were no significant differences in the NTCP values between the two approaches for the serial organs.

**Conclusions:**

Our results showed that the BBTP approach could be a potential alternative approach to the DVTP approach for NPC.

## Introduction

There is a high incidence of nasopharyngeal carcinoma (NPC) in the South China Region especially in the Guangdong province. NPC is also common in the southeast Asia such as Indonesia [1], [2]. Radiation therapy (RT) is one of the major treatment modality for nasopharyngeal carcinoma (NPC). Currently, intensity-modulated radiation therapy (IMRT) is one of the most common and effective radiotherapy treatment techniques for NPC [3]–[9]. IMRT is based on the use of a number of radiation beams with nonuniform intensities coming from different directions. The nonuniform fluence map of each beam is optimized by a computer method to produce a composite dose distribution criteria predefined by the planner [10]. Physical quantities such as dose and dose-volume (DV) parameters are usually used for IMRT fluence optimization and plan evaluation in the clinical setting. IMRT optimization using biological related approach (BBTP) that employs biological parameters such as tumor control probability (TCP), normal tissue complication probability (NTCP) and equivalent uniform dose (EUD) has recently been proposed for IMRT planning [11]. Biological related parameters have more direct correlation with treatment outcome than DV based parameters. It is believed that BBTP can provide more flexibility during optimization and may produce more optimal treatment plans. Some investigations have reported that when compared to using DV-based parameters, the use of biological or a combination of both biological and DV-based parameters in IMRT planning could produce better sparing of critical organs without compromising target coverage [12]–[18]. It has been shown that IMRT optimization using purely biological parameters would result in highly inhomogeneous target dose distributions with an undesirable amount of hot spots [12], [13], [18]. To improve the target dose homogeneity and conformity, some investigators have proposed to combine the use of physical dose parameters with biological based models for optimization [11], [13], [16]–[18]. For example, Wu et al. proposed the use of the generalized EUD optimization followed by an DV-based optimization using a gradient technique to fine-tune the dose volume histogram (DVHs), while Das et al. proposed to incorporate biological optimization after DV constrained optimization [13], [18].

The Eclipse System (Varian Medical Systems, Palo Alto, CA) is one of the commercially available treatment planning systems (TPS) that allows the users to perform BBTP using biological related cost functions together with some physical constraints for fluence optimization. Most of the previous evaluation studies on BBTP were performed using in-house developed TPS systems. Evaluation using two other commercial system, the MONACO (CMS/Elekta, St. Louis, MO) and PINNACLE (Philips Medical Systems, Andover, MA) systems have been reported for various diseases [16], [17]. However, different planning systems provide different approaches of biological related optimization models. Extensive experiences for using the Eclipse System for BBTP have not been reported. The performance of a commercially available fluence optimization algorithm should be thoroughly tested before it can be used extensively for clinical cases. The main objective of our investigation was to assess the performance of the current commercially available BBTP optimization approach installed in the Eclipse system as compared to its conventional dose/dose volume based approach (DVTP) using NPC. Delivering a curative dose to the tumor while sparing the surrounding critical organs for NPC is one of the most challenging tasks for IMRT planning. The tumor is usually located near a relatively larger number of critical normal organs when compared to other diseases. These include the brain stem, spinal cord, parotid and the optic structures. Target dose homogeneity is also difficult to maintain in a NPC IMRT plan as there is a lot of tissue inhomogeneity inside the planning target volume, including air, bone and soft tissues. In addition, the planning also involves the prescription of multiple dose levels simultaneously to different target volumes. Planning for this disease would be a good demonstration on the performance of the system. Most plan evaluations of previous studies for biologically based IMRT optimization methods were performed using conventional physical dose quantities, while both biological indices and physical parameters were employed in the current study for plan quality evaluation. For NPC cases, the plan complexity level varies with the staging of the disease. The target volumes of advanced cases are usually larger and thus closer to its surrounding critical organs, representing a more difficult planning task. The estimation of TCP values also varies with staging. Therefore, plan evaluations for early and advanced cases were separated into two categories in this study.

## Methods

The personal information of patients was anonymized before and during the study, and no patient information was extracted from patient charts and/or utilized in this study. None of the patient's personal information was presented in the manuscript. Neither gender nor age was disclosed. The Computed Tomography (CT) data of patients were merely used for the dosimetric study. We made use of different patients' tumor and normal organ configurations to evaluate the power of two different optimization algorithms in IMRT planning based on dose distributions calculated on the CT image data by the treatment planning system. This study was to compare the pros and cons of the recently developed biological optimization algorithm to those of the conventional dose/dose-volume optimization algorithm. This was a retrospective study. Patient treatment outcomes were not involved.

20 NPC patients who were treated with IMRT plans optimized using the DVTP approach were re-planned retrospectively using the BBTP approach on the same platform using the Eclipse system. Patients selected were with tumors that range from stage I to IV (1997 UICC/AJCC stage classification). Ten of them were early stage (stage I or II) cases and the rest were advanced (stage III or IV) and complex cases. The authors decided to evaluate these two categories of cases separately because the targets of advanced cases usually involved parts or even the whole of the sphenoid sinus and infiltrated a considerable amount of clivus, making the sparing of optical structures and brain stem more difficult than that for the early stage cases. All IMRT plans were generated using 6 MV photon beams and modulated with 120 multileaf collimator from a linear accelerator (Clinac 23EX or 6EX, Varian Medical Systems). All the plans were generated using the Eclipse version 10.0 (Varian Medical Systems Inc., Palo Alto, CA) incorporated with the biological planning tools developed by the RaySearch Laboratories (Sveavägen 25 111 34 Stockholm Sweden). All dose calculations were performed with the anisotropic analytical algorithm (AAA) using a calculation grid of 2.5 mm. AAA must be chosen for dose calculations in the current study because the biological optimization option for the current version of Eclipse system used the same beam configuration data as AAA. In order to avoid the variation of IMRT plan quality due to operator skill and experience, all IMRT plans were high quality plans generated by one single experienced planner following the institutional planning conventions.

### Treatment planning

The same photon beam settings including number and orientations of beams, beam energy and isocenter position used for each DVTP based plan were used for its corresponding BBTP based plan. This is to eliminate the differences in plan quality due to the variation in radiation beam parameters. The IMRT plans were created using 9 to 11 evenly distributed coplanar fields. Multiple dose levels prescribed to the planning target volumes (PTVs) were achieved with the simultaneous integrated boost technique [19]. The dose to the PTV_70_ that included the primary gross tumor volume and the nodal gross tumor volume encompassed positive lymph nodes was set to 70 Gy. The dose to PTV_60_ that included the high-risk clinical target volume (CTV) and the nodal CTV was set to 60 Gy. The dose to PTV_54_ that included the low risk CTV was set to 54 Gy. According to our local clinical practice for DVTP, the optimization goal was to ensure at least 95% of the PTVs to receive the prescribed dose, while limiting the doses to organs at risk (OARs) including brain stem, spinal cord, optic nerves, optic chiasm, lens and parotid glands according to the constraints listed in table 1. The optimization dose priorities were consistent for all cases. Giving enough dose coverage to PTVs and limiting the maximum doses to brain stem, spinal cord and optic nerves were given the highest priority, followed by reducing the dose to parotid glands. Lower priorities were given to the other critical organs. To improve the target conformity, an optimization criterion was also assigned to an organ representing normal tissues (avoid_normal), which was defined as the body volume in the CT data set minus the PTV leaving a 3 mm gap. For early stage cases, the prescription doses to targets were given in 35 daily fractions. For the advanced cases, the prescription doses were given in 33 daily fractions. The constraints listed in table 1 were also used as the plan acceptability criteria when performing the BBTP.

**Table 1:** Constraints to OARs.

OAR	Constraints	
	Maximum dose	Dose volume
Brain stem	54 Gy	less than 1% should receive up to 60
		Gy if maximum dose cannot be
		achieved
Spinal cord	45 Gy	less than 1% or 1 cc should receive
		up to 50 Gy if maximum dose cannot
		be achieved
Optic nerves/chiasm	54 Gy	less than 1% should receive up to 60
		Gy if maximum dose cannot be
		achieved
Parotid glands		mean dose ≤26 Gy or <50% volume
		to exceed 30 Gy if mean dose cannot
		be achieved
Eyes	45 Gy	Mean dose <35 Gy if maximum dose
		cannot be achieved
Lens	6 Gy	less than 1% should receive up to 10
		Gy if maximum dose cannot be
		achieved

### Biological Optimization in Eclipse

Biological optimization in the Eclipse system is not a built-in option, but is an add-on software component developed by RaySearch Laboratories. It can produce an ideal set of fluence maps for the IMRT treatment plan based on radiobiological models using a combination of biological and physical criteria. The plans created by biological optimization can be evaluated using the build-in dose volume analysis tool and/or the add-on biological evaluation tool. The objective functions which are to be maximized or minimized during the optimization include the TCP Poisson-LQ and the NTCP Poisson-LQ [20]–[26]. The TCP and NTCP Poisson-LQ could be obtained either based on the Linear Quadratic (LQ) cell survival model or equivalently the Linear dose-response model with the Equivalent dose in 2-Gy fractions (EQD_2_). The relative seriality model proposed by Källman et al. was used for NTCP Poisson-LQ. A high value of seriality would be used for serial organs that were sensitive to high local doses even though the mean doses were low, while a lower value of seriality would be used for parallel organs that were less sensitive to local high doses, but still affected by high and low doses [22]. When both TCP functions for targets and NTCP functions for OARs are defined for a task, the probability for complication free tumor control (P+) would become the objective function. This was an attempt to combine the individual TCPs and NTCPs into a single measure of the plan quality. Prior to the biological optimization, at least one TCP has to be defined for the target structure. In addition to the objective functions, a number of optional constraints could be defined by the users and were listed in table 2. The goal of optimization was to produce the best value of the objective function without violating any of the constraints.

**Table 2:** Details of optional constraints available for targets and OARs.

Constraint	Parameters to specify	Description of the constraint
	*For target structures*	
Min Dose	Dose level	It is achieved when all pixels of the
		structure have minimum dose greater
		than or equal to the specified dose level
Max Dose	Dose level	It is achieved when all pixels of the
		structure have maximum dose less than
		or equal to the specified dose level
Min DVH	Dose level and percentage of	It is achieved when at least the specified
	volume	percentage of volume receives at least
		the specified dose level
Max DVH	Dose level and percentage of	It is achieved when no more than the
	volume	specified percentage of volume receives
		at most the specified dose level
Min EUD	Dose level and parameter A*	It is achieved when the equivalent
		uniform dose (EUD) value is at least the
		specified dose level
Max EUD	Dose level and parameter A	It is achieved when the EUD value is at
		most the specified dose level
Target EUD	Dose level and parameter A	It is achieved when the EUD value is
		equal to the specified dose level
Uniformity	Standard deviation (std.dev %)	It is achieved when the variation of dose
constraint		within the structure is less than the
		specified std. dev %
	*For OARs*	
Max Dose	Dose level	same meaning as for target structure
Max DVH	Dose level and percentage of	same meaning as for target structure
	volume	
Max EUD	Dose level and parameter A	same meaning as for target structure
Target	High dose level, low dose	It describes the variation of dose with
conformance	level and low dose distance	distance from the target. It is achieved
		when the dose at the target border
		decreases from the high dose level to the
		low dose level through the low dose
		distance.

*For A<1, higher weighting will be given to low doses so as to avoid the occurrence of cold spots. For A>1, higher weighting will be given to high doses so as to avoid the occurrence of hot spots. For A = 1, equal weighting is given to cold and hot spots.

The biological functions and additional constraints that were used for the biological optimization of the NPC cases were listed in table 3. The Poisson TCP was only applied to PTV_70_ that included the tumor bed. The target EUD was used for each individual target to achieve multiple dose levels. In additions, Max Dose and Uniformity constraint were also used to reduce hot spots and improve the target dose heterogeneity. In order to produce target conformities that were comparable to those accepted in our local clinical practice, a Max Dose constraint was assigned to the normal tissues represented by avoid_normal. Using NTCP alone usually could not achieve desirable doses that were below the clinically acceptable dose tolerance levels for certain organs, but the use of Max Dose and Max EUD constraints helped further reduce the doses to OARs.

**Table 3:** The biological functions and additional constraints that are selected for optimization of the NPC patient plans.

Structure	Functions used
PTV_70_	(1)TCP Poisson LQ with D_50_ = 57.40 Gy, γ = 6.3, α/β = 10 Gy
	(2)Target, EUD
	(3)Max Dose
	(4)Uniformity constraint
PTV_60_-PTV_70_	(1)Target, EUD
	(2)Uniformity constraint
PTV_54_	(1)Target, EUD
	(2)Uniformity constraint
Brain stem	(1)NTCP Poisson LQ with D_50_ = 65.10 Gy, γ = 2.4, α/β = 3 Gy,
	s = 1; endpoint: necrosis/infarction
	(2)Max EUD
	(3)Max Dose
Spinal cord	(1)NTCP Poisson LQ with D_50_ = 68.60 Gy, γ = 1.9, α/β = 3 Gy,
	s = 4; endpoint: myelitis necrosis
	(2)Max EUD
	(3)Max Dose
Optic chiasma	(1)NTCP Poisson LQ with D_50_ = 65.00 Gy, γ = 2.3, α/β = 3 Gy,
	s = 1; endpoint: blindness
	(2)Max Dose
Optic nerve	(1)NTCP Poisson LQ with D_50_ = 65.00 Gy, γ = 2.3, α/β = 3 Gy,
	s = 1; endpoint: blindness
	(2)Max Dose
Eye retina	(1)NTCP Poisson LQ with D_50_ = 65.00 Gy, γ = 1.8, α/β = 3 Gy,
	s = 1; endpoint: blindness
Lens	(1)NTCP Poisson LQ with D_50_ = 18.00 Gy, γ = 1.2, α/β = 3 Gy,
	s = 1; endpoint: cataract
Parotid	(1)NTCP Poisson LQ with D_50_ = 46.00 Gy, γ = 1.8, α/β = 3 Gy,
	s = 1; endpoint: xerostomia
	(2)Max EUD
Avoid_normal	(1)Max Dose

The Eclipse system allows the users to adjust the parameters of the TCP and NTCP functions in the biological optimization template such as the D_50_, γ and α/β values. The BBTP plan quality can be varied by adjusting these values. The plan comparison results reported in the current study are confined to values of the biological parameters as specified in table 3.

### Comparison of plan quality using physical and biological quantitative indices

Comparison of plan quality between BBTP and DVTP plans were based on quantitative physical dose and biological indices estimated from the dose volume histogram (DVH). The DVH curves were derived from the three dimensional dose distributions calculated on the CT images. The coverage of PTVs of BBTP and DVTP based plans were evaluated by comparing the target volumes receiving 95% of the prescribed dose (V_95%_). The maximum doses represented by the doses received by 2% of the target volumes (D_2%_), the minimum doses represented by 98% of the target volumes (D_98%_) and the mean doses were reported. The degree of target conformity of the plans was evaluated using the confirmation number, CN. This was defined as the product of V_T,ref_/V_T_ and V_T,ref_/V_ref_, where V_T,ref_ represented the volume of the target receiving a dose equal to or greater than the reference dose; V_T_ represented the physical volume of the target, and V_ref_ represented the total tissue volume receiving a dose equal to or greater than the reference dose [27]. The prescription dose was used as the reference dose to compute the CN. The first fraction represents the target coverage, while the second fraction represents the amount of healthy tissue receiving a dose greater than or equal to the reference dose. Its value ranges from 0 to 1, where 1 represents ideal conformity and 0 represents total absence of conformity. The target dose homogeneity (HI) was expressed in terms of the ratio (D_2%_–D_98%_)/D_50%_, where D_50%_, is the minimum doses represented by 50% of the target volumes [28]. A lower HI value indicates a more homogeneous target dose. The Biological Plan Evaluation plug-in software package in Eclipse was used to compute the TCP based on the Poisson model with the parameters: D_50_ = 57.4 Gy, γ = 6.3, α/β = 10 Gy for stage 1 and 2 cases, D_50_ = 60.2 Gy, γ = 4.2, α/β = 10 Gy for T3 cases and D_50_ = 67 Gy, γ = 3.0, α/β = 10 Gy for stage 4 cases [25].

For OARs, the maximum doses to one percentage volumes (D_1%_) and five percentage volumes (D_5%_) of spinal cord, brainstem, optic chiasm and optic were reported. The mean doses for parotid glands, eyes and lens were reported. The volume of parotid glands receiving more than 30 Gy (V_30Gy_), 40 Gy (V_40Gy_) and 50 Gy (V_50Gy_) were analyzed. The NTCP based on the Poisson model of each OAR with parameters and endpoints the same as those listed in table 3 was also assessed [26].

The Wilcoxon matched-pair signed rank test was used to compare the results between BBTP based and DVTP based plans. The threshold for statistical significance was p ≤0.05. All statistical tests were two-sided, and all analyses were performed using the Statistical Package for Social Sciences (SPSS, Chicago, IL) software, version 11.0. The Wilcoxon signed rank test assumed that the patients selected were randomly taken from a population, and the distribution of the paired differences was symmetric.

## Results

### Targets of early stage NPC

**Figure 1:**
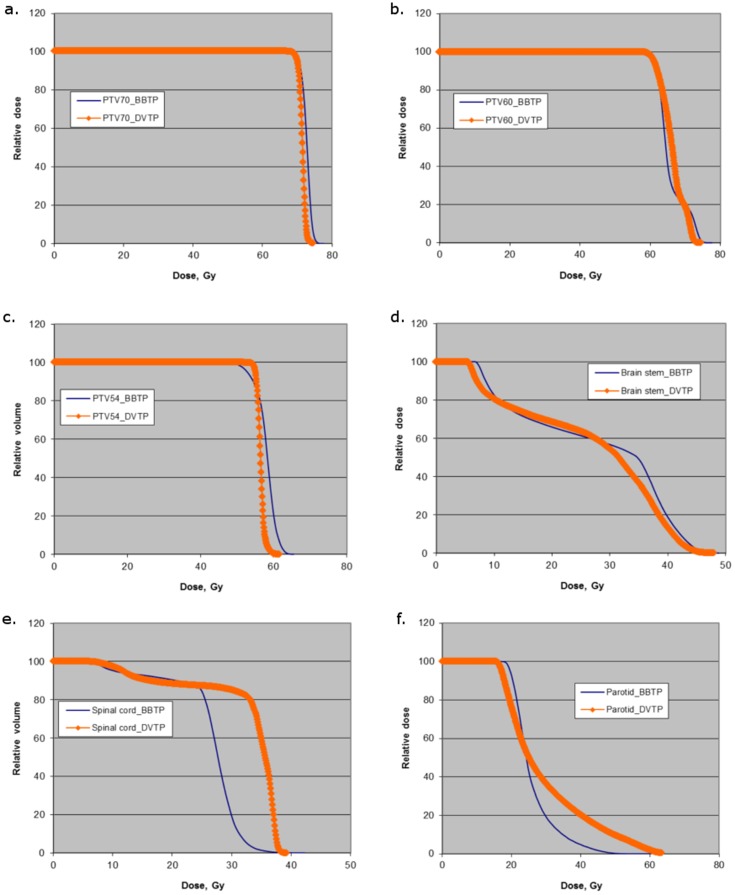
Comparison of the dose volume histograms between BBTP and DVTP plans for various targets including (a) PTV_70_, (b) PTV_60_, (c) PTV_54_ and various OARs including (d) brain stem, (e) spinal cord, (f) parotid of a typical early stage NPC patient.

As shown in table 4, for both BBTP plans and DVTP plans, more than 99% of the target volumes (PTVs) of early stage NPC received at least 95% of the prescribed dose. For PTV_70_, BBTP plans achieved similar conformity but an inferior dose homogeneity when compared to DVTP plans. About 15% of the PTV_70_ volumes received a dose greater than or equal to 107% of the prescribed dose for BBTP based plans, while it was 0% for DVTP based plans. The average maximum dose for BBTP plans was about 3% higher. More hot spots were found in BBTP plans even though more than one additional physical constraints were added to control the dose homogeneity during the optimization process. The TCP values for both BBTP and DVTP plans were more than 98% and the values were comparable with each other. For PTV_60_, BBTP plans achieved slightly inferior conformity and dose homogeneity when compared to DVTP plans, with BBTP producing about 1.5% lower mean doses to PTV_60_ than the DVTP plans. Figure 1 (a) to (c) shows the DVH curves of PTV_70_, PTV_60_ and PTV_54_ for a typical early stage patient.

**Table 4:** Summary of biological and physical evaluation results for targets averaged over 10 early stage NPC patients.

Parameter/function	BBTP	DVTP	p†
**PTV_70_**			
TCP Poisson	98.91±1.50	98.64±1.73	∼
Mean dose, Gy	73.28±0.76	72.02±0.25	*
Maximum dose, Gy	75.56±0.88	73.44±0.35	*
Minimum dose, Gy	69.62±0.44	70.05±0.27	*
V_110%_, %	0.39±0.69	0.00±0.00	∼
V_107%_, %	15.71±16.36	0.00±0.00	∼
V_95%_, %	99.83±0.31	100.00±0.01	∼
CN	0.84±0.03	0.85±0.02	∼
HI	0.08±0.01	0.05±0.01	*
**PTV_60_**			
Mean dose, Gy	65.74±0.61	66.71±0.34	*
Maximum dose, Gy	74.50±1.52	72.93±0.35	∼
Minimum dose, Gy	59.45±0.54	60.72±0.43	*
V_95%_, %	99.49±0.27	99.97±0.03	*
CN	0.81±0.05	0.85±0.03	∼
HI	0.23±0.03	0.18±0.01	*
**PTV_54_**			
Mean, Gy	58.19±0.89	56.50±0.32	*
V_95%_, %	99.57±0.73	99.89±0.13	∼

† The symbol “*” indicates that the mean difference between the pair was proved to be statistically significant with a p-value ≤0.05 and the symbol “∼” indicates statistically insignificant result.

### Targets of advanced stage NPC

Table 5 summarized the average results for targets for advanced stage NPC patients. It could be seen that, similar to those for early-stage diseases, more than 99% of the PTVs received 95% of the prescribed dose. In terms of target conformity and dose homogeneity, similar trends were observed as described in the previous section. However, for this category of cases, even more hot spots were generated by the BBTP plans. For PTV_70_, the values of V_107%_ reached 41% for BBTP based plans, while it was only 0.8% for DVTP based plans. The average maximum dose and mean dose of BBTP plans were 4.4% and 2.7% higher than those of the DVTP plans, respectively. The average TCP values for PTV_70_ of advanced cases were lower than those of the early stage cases, which were 88.9% and 86.2% for the BBTP and DVTP plans, respectively. For the advanced cases, the higher doses delivered to the PTV_70_ produced using biological optimization resulted in higher TCP values.

**Table 5:** Summary of biological and physical evaluation results for targets averaged over 10 advanced stage NPC patients.

Parameter/function	Biological based optimization	Dose volume based optimization	p†
**PTV_70_**			
TCP Poisson	88.95±9.61	86.19±11.00	*
Mean dose, Gy	74.26±0.55	72.31±0.40	*
Maximum dose, Gy	77.14±0.68	73.91±0.73	*
Minimum dose, Gy	69.57±1.06	70.20±0.29	∼
V_110_, %	3.57±2.37	0.03±0.08	*
V_107_, %	41.14±15.56	0.79±1.55	*
V_95_, %	99.78±0.29	99.97±0.04	∼
CN	0.86±0.04	0.86±0.02	∼
HI	0.10±0.02	0.05±0.01	*
**PTV_60_**			
Mean dose, Gy	67.06±0.84	67.55±0.94	∼
Maximum dose, Gy	76.35±0.60	73.51±0.71	*
Minimum dose, Gy	60.04±1.34	61.23±0.35	*
V_95_, %	99.57±0.17	99.98±0.02	∼
CN	0.80±0.04	0.85±0.01	∼
HI	0.25±0.01	0.18±0.01	*
**PTV_54_**			
Mean, Gy	58.26±1.13	56.88±0.32	*
V_95_, %	99.00±1.20	99.41±0.87	∼

† The symbol “*” indicates that the mean difference between the pair was proved to be statistically significant with a p-value ≤0.05 and the symbol “∼” indicates statistically insignificant result.

### OARs of early stage NPC

**Figure 2:**
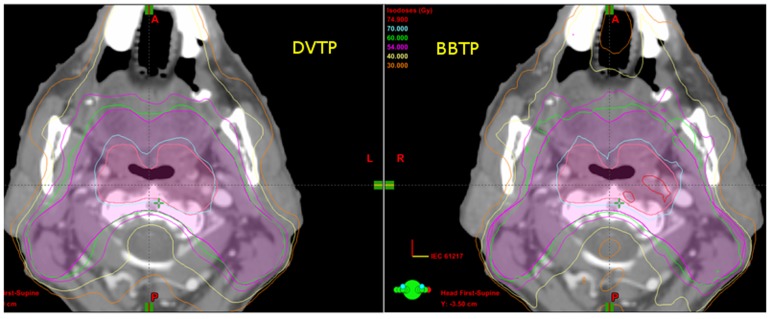
One of the axial computed tomography (CT) slices comparing the isodose curves between the BBTP plan and the DVTP plan of a typical NPC patient.

Table 6 summarized the average results for OARs of early stage NPC patients. For all the serial organs including brain stem, spinal cord, optic nerve and optic chiasma, there were no significant differences in the values of D_1%_ and NTCP between BBTP plans and DVTP plans. All D_1%_ values were lower than the tolerance dose level and the NTCP values approach to zero for both types of plans. The D_5%_ for spinal cord of BBTP plans was 2.8 Gy lower than that of DVBP plans and was statistically significant. This could also be reflected by the DVH curve shown in figure 1 (e). For parotid glands, the average NTCP value was 21.3% for DVTP plans, while it was only 8.0% for BBTP plans, indicating a significant reduction in the NTCP value with the use of biological optimization. The mean dose and V_30Gy_ of BBTP plans were slightly lower when compared to DVTP plans. However, V_40Gy_ and V_50Gy_ (volumes receiving higher doses), were significantly reduced from 25.0% to 10.8% and 13.3% to 4.0%, respectively, when using BBTP plans rather than DVTP plans. The DVH curve of figure 1 (f) also showed that a significantly lower volume of the parotid glands under the BBTP plans received the higher dose values. The isodose lines shown in figure 2 shows that the dose fall off near the parotid glands was steeper for the BBTP plan than that for the DVTP plan of a typical patient. For eye retina and lens, the average mean doses for DVTP plans were 1.8 and 1.9 Gy lower than those of the BBTP plans, respectively, inducing no significant difference in NTCP values.

**Table 6:** Summary of biological and physical evaluation results for OARs averaged over 10 early stage NPC patients.

Parameter/function	Biological based optimization	Dose volume based optimization	p†
**Brain stem**			
NTCP Poisson	0.01±0.01	0.01±0.01	∼
D_1%_, Gy	48.41±1.20	47.92±1.83	∼
D_5%_, Gy	46.04±1.17	45.37±2.21	∼
**Spinal cord**			
NTCP Poisson	0.00±0.01	0.00±0.01	∼
D_1%_, Gy	38.97±1.76	40.17±1.37	∼
D_5%_, Gy	36.50±2.10	39.28±1.36	*
**Optic nerve**			
NTCP Poisson	0.00±0.00	0.00±0.00	∼
D_1%_, Gy	14.31±5.36	15.19±9.29	∼
D_5%_, Gy	12.69±4.48	13.06±7.74	∼
**Optic chiasma**			
NTCP Poisson	0.00±0.00	0.00±0.00	∼
D_1%_, Gy	11.80±3.40	14.25±9.93	∼
D_5%_, Gy	11.23±3.12	12.85±7.95	∼
**Eye retina**			
NTCP Poisson	0.00±0.00	0.00±0.00	∼
Mean, Gy	7.24±2.17	5.42±1.39	*
**Lens**			
NTCP Poisson	0.02±0.04	0.00±0.00	∼
Mean, Gy	5.50±1.56	3.62±0.29	*
**Parotid**			
NTCP Poisson	8.04±5.84	21.31±8.28	*
Mean, Gy	30.30±2.25	31.26±1.39	∼
V_30Gy_, %	39.59±11.83	41.35±2.64	∼
V_40Gy_, %	10.78±6.11	24.96±3.75	*
V_50Gy_, %	4.00±3.77	13.34±5.33	*

† The symbol “*” indicates that the mean difference between the pair was proved to be statistically significant with a p-value ≤0.05 and the symbol “∼” indicates statistically insignificant result.

### OARs of advanced stage NPC

As shown in table 7, similar to results for the early stage cases, there were no significant differences observed for the dose values and NTCP values between BBTP plans and DVTP plans for all the serial organs. The averaged NTCP values were smaller than 0.1% for brain stem and spinal cord, while those for optic nerve and chiasma were smaller than 4.0% for both types of plans. For parotids, the average NTCP value was 24.4% for DVTP plans, while it was only 7.9% for BBTP plans. There were no significant differences in the parotid mean dose and V_30Gy_ between the two types of plans. The V_40Gy_ and V_50Gy_ for BBTP plans were significantly reduced from 25.7% to 9.5% and 14.1% to 2.9%, respectively, when compared to those of the DVTP plans. For eye retina and lens, the average mean dose for DVTP plans was significantly lower than that of the BBTP plans. There was no significant difference in NTCP values for eye retina and the values of both types of plans were very low and close to 0.01%. For lens, the average NTCP value for DVTP plans was 13.4% lower than that of the BBTP plans.

**Table 7:** Summary of biological and physical evaluation results for OARs averaged over 10 advanced stage NPC patients.

Parameter/function	Biological based optimization	Dose volume based optimization	p†
**Brain stem**			
NTCP Poisson	0.04±0.03	0.06±0.04	∼
D_1%_, Gy	48.73±1.21	50.17±1.27	∼
D_5%_, Gy	45.96±1.63	47.57±1.70	∼
**Spinal cord**			
NTCP Poisson	0.06±0.10	0.03±0.04	∼
D_1%_, Gy	39.51±1.64	39.46±0.92	∼
D_5%_, Gy	37.23±1.93	38.48±0.94	∼
**Optic Nerve**			
NTCP Poisson	2.75±2.79	3.45±5.48	∼
D_1%_, Gy	54.83±4.37	54.31±4.72	∼
D_5%_, Gy	52.15±5.32	52.56±5.57	∼
**Optic chiasma**			
NTCP Poisson	3.79±4.50	2.40±2.31	∼
D_1%_, Gy	54.78±5.25	53.43±6.42	∼
D_5%_, Gy	53.19±6.09	51.73±7.00	∼
**Eye retina**			
NTCP Poisson	0.01±0.02	0.01±0.03	∼
Mean, Gy	17.63±5.16	9.52±3.03	*
**Lens**			
NTCP Poisson	17.02±23.48	3.62±11.43	∼
Mean, Gy	15.06±6.35	5.27±1.57	*
**Parotid**			
NTCP Poisson	7.92±8.73	24.42±12.82	*
Mean, Gy	30.61±2.27	31.56±4.03	∼
V_30_, %	41.90±9.09	41.67±9.27	∼
V_40_, %	9.50±6.48	25.65±7.61	*
V_50_, %	2.93±4.66	14.14±7.53	*

† The symbol “*” indicates that the mean difference between the pair was proved to be statistically significant with a p-value ≤0.05 and the symbol “∼” indicates statistically insignificant result.

## Discussion

Investigations from previous studies using other treatment planning systems showed that BBTP plans would in general produce lower doses to most OARs while maintaining similar target coverage [12], [13], [16], [17]. Our results using the Eclipse system focused on NPC cases showed that the use of biological related model based optimization might not be beneficial to all/most OARs. It was observed in the current study that BBTP plans produced better sparing of the parotid glands, while similar sparing of most serial organs and inferior sparing of eye retina and lens when compared to DVTP plans.

The most obvious benefit that could be observed in NPC cases using the Eclipse biological optimization module over the conventional dose/dose volume based optimization was the better sparing of the parotid glands. The Poisson NTCP values estimated for xerostomia was also significantly reduced. Although the mean doses of the parotids were not significantly reduced, the use of Max EUD as one of the constraints effectively reduced the volume of parotids receiving doses beyond 40 Gy. The degree of xerostomia strongly depends on the radiation dose and the volume of the salivary gland being irradiated. The salivary function can be recovered over time even with doses up to 40–50 Gy. However, higher doses to a large volume of parotid gland might lead to irreversible and permanent xerostomia [5], [29], [30]. Therefore, a significant reduction of volume receiving doses above 40 Gy would likely be beneficial to the patients. However, the current study was confined to comparisons based on dose distributions of the treatment plans. Further investigations are required in the future to evaluate the clinical benefits of using the BBTP approach.

Since the parotid glands were in close proximity to the PTV_60_, their better sparing from high doses were achieved at the expenses of lowering the mean doses to PTV_60_. Although the target coverage represented by V_95%_ of PTV_60_ was not changed, tables 4 and 5 showed that the average mean dose of PTV_60_ for BBTP plans was approximately 1 Gy lower than those for DVTP plans. This could also be reflected from the DVH curves shown in figure 1b. The eye retina and lens did get higher doses by using biological optimization in the current study. The reason might be that these two were the only normal organs for which none of the physical constraints were used during optimization, and the Poisson NTCP function was the only objective function used. Adding physical constraints like Max EUD and Max Dose would help to decrease the doses. However, for eye retina, this might be practically irrelevant as the NTCP values to cause blindness approached zero for both types of plans. On the other hand, the doses to lens to cause cataract might not be a good indicator to compare the two optimization methods as cataract could be corrected by surgery and it was sometimes debatable to consider it as clinically relevant toxicity.

It has been reported that by solely using TCP/EUD as the objective function would result in highly inhomogeneous target doses. TCP/EUD is less sensitive to hot spots as they help maximize target-cell kill. It is believed that adding some physical dose cost functions during the optimization process might help to improve the target dose homogeneity [5]. In this study, two additional physical constraints were used, namely, the Uniformity constraint and the Max Dose constraint. The Uniformity constraint was set to 1% and the Max Dose was set to 72 Gy or lower for PTV_70_. Although the amount of hot spots and the maximum doses within targets were substantially reduced by adding these physical constraints, the HI, V_110%_ and V_107%_ values still reflected that BBTP plans produced inferior target dose homogeneity with more hot spots when compared to the DVTP plans. During the dose volume based optimization, hot spots found within the target volumes could be contoured as virtual organs. They could then be effectively removed by setting upper dose limits with desired priority after several repeated optimization cycles. The authors had attempted similar strategy for biological optimization but it was not effective. This might be due to the fact that achieving the desired TCP and Target EUD were inherently given higher priorities compared to the physical dose constraints during optimization. The current BBTP approach does not allow the users to specify the priority of physical constraints like Max Dose and Uniformity. It is highly desirable if priority could be given to these constraints so that the users could have a better control of the target dose homogeneity.

The advantage of providing better sparing of parotid glands was counter-balanced by producing more hot spots within the targets. However, whether the appearance of hot spots within the target volumes is clinically undesirable or not is debatable. Hot areas within the tumor bed were commonly found when using stereotactic radiotherapy and brachytherapy. Hot areas and higher maximum doses could be beneficial to advanced stage diseases if they were located within the gross tumor volume, as reflected from the higher TCP values for the BBTP plans observed in the advanced NPC cases in the current study. Unfortunately, some hot spots might be found at undesirable locations close to or in the skull base. According to our clinical practice, not all vessels and nerves close to or in the skull base are contoured as OARs. It is therefore easy to miss them during plan evaluation. Hot spots in the skull base can inadvertently cause radiation damage to structures like carotid artery pseudoaneurysm and hypoglossal nerves palsies. They might also cause bone necrosis if the dose is too high. Planners and oncologists need to be very careful in assessing the hot spots during plan evaluation. As shown in tables 4 and 5, it was found that less than 20% of the PTV_70_ received more than 110% of the prescribed dose in all the BBTP plans generated in the current study. These results at least did not violate the protocol designed by the Radiation Therapy Oncology Group (RTOG 0225) of the American College of Radiology (ACR).

Comparing with dose/dose volume based optimization, biological optimization was simpler and more effective. Based on our usual clinical practice using dose/dose volume based optimization, a large number of virtual organs were required for avoidance to reduce the dose outside the PTVs and contoured as hot and cold areas to improve target dose homogeneity. It is common to repeat the optimization cycles more than 5 to 6 times for adjusting the dose distributions through the trial and error approach. When using the biological optimization, the only virtual organ used was the normal tissue as listed in table 3. All the objective functions and physical constraints used were those listed in table 3. The total number of parameters used and the number of optimization cycles required were smaller than half of those used in dose/dose volume based optimization.

## Conclusions

The use of Eclipse biological related model for IMRT optimization in NPC produced comparable target coverage, target conformity and improved sparing of parotid glands with lower NTCP values when compared to physical dose based optimization operating on the same platform. For the primary target, BBTP plans produced comparable TCP values for early stage NPC cases and improved TCP values for advanced stage NPC cases compared to DVTP plans. The doses to serial organs and their corresponding NTCP values were comparable between the two approaches. The BBTP plans produced inferior target dose homogeneity with occurrence of mainly hot spots rather than cold spots. The hot spots were mainly located within the primary target volume with maximum doses of about 110%. This might be considered as clinically acceptable depending on the local practice of individual centers and the location of the hot spots. The use of biological based optimization combined with physical constraints can be a potential alternative to the conventional dose/dose volume based optimization for NPC cases. However, hot spots are sometimes considered as undesirable in NPC especially when they are located close to the skull base. There is still room for further improvement in the current BBTP approach in terms of improving target dose homogeneity.
